# Supplementary figures and images for: The uncharacterized protein FAM47E interacts with PRMT5 and regulates its functions
Source: Life Sci Alliance. 2020 Dec 29;4(3):e202000699. doi: 10.26508/lsa.202000699 (PMC7772775; doi:10.26508/lsa.202000699)

**Figure 1F:** Endogenous IP: FAM47E: Uncropped Images

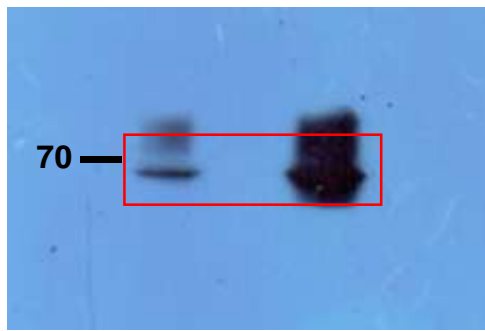

IB:  $\alpha$ -PRMT5

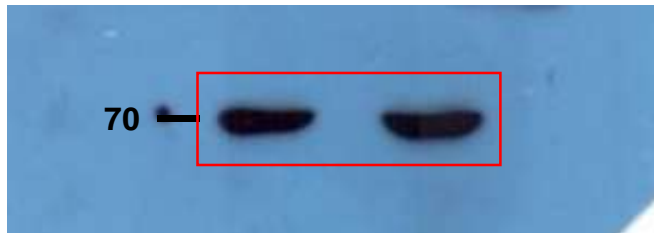

WCE:  $\alpha$ -PRMT5

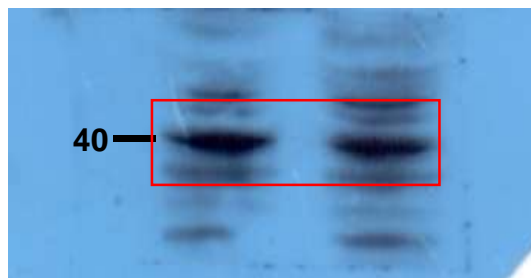

WCE:  $\alpha$ -FAM47E

Supplement: Supplementary file 1 [file LSA-2020-00699_SdataF1.pdf]

**Figure S3: IP: GFP-Trap: Uncropped Images**

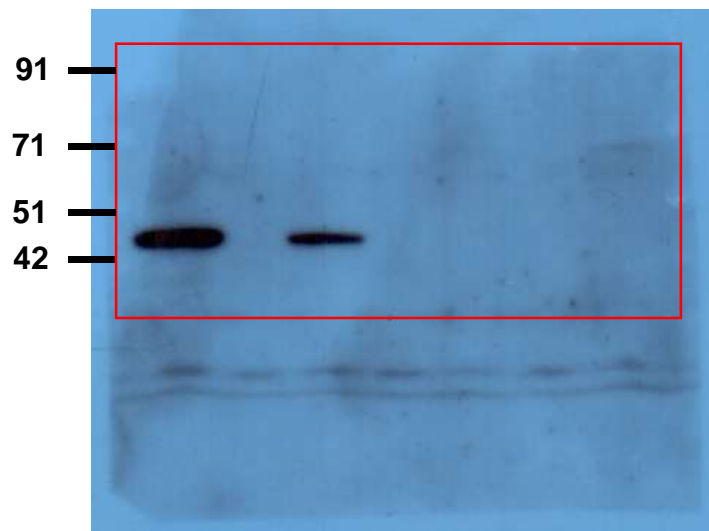

**IB:  $\alpha$ -SYM10**

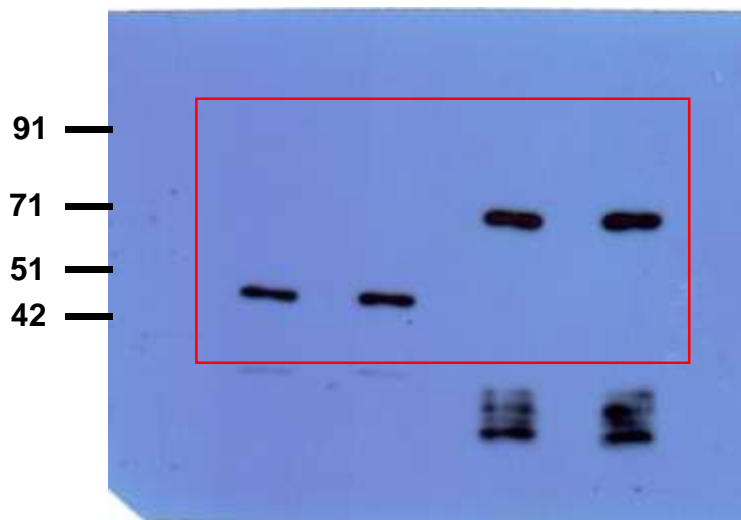

**Input:  $\alpha$ -GFP**

Supplement: Supplementary file 2 [file LSA-2020-00699_SdataFS3.pdf]
